# Epstein-Barr Virus Infection and Sporadic Breast Cancer Risk: A Meta-Analysis

**DOI:** 10.1371/journal.pone.0031656

**Published:** 2012-02-21

**Authors:** Qiang Huo, Ning Zhang, Qifeng Yang

**Affiliations:** Department of Breast Surgery, School of Medicine, Shandong University, Qilu Hospital, Ji'nan, Shandong, People's Republic of China; Health Canada, Canada

## Abstract

**Background:**

A large number of epidemiological studies have evaluated the association between Epstein-Barr virus infection and breast carcinoma risk but results have been inconsistent.

**Methodology:**

Research using the polymerase chain reaction technique for detecting the Epstein-Barr virus was selected; 24 studies and 1535 cases were reviewed. Information on the study populations, sample types, publication calendar period and histological types of breast carcinoma were collected. An unconditional logistic regression model was used to analyze potential parameters related to the Epstein-Barr virus prevalence. A Kappa test was used to evaluate the consistency in detecting different Epstein-Barr virus DNA regions. Nine studies that included control groups and 1045 breast cancer cases were adopted in this meta-analysis.

**Conclusions:**

We found that 29.32% of the patients with breast carcinoma were infected with the Epstein-Barr virus. The prevalence of Epstein-Barr was highest in Asia (35.25%) and lowest in the USA (18.27%). Statistical analysis revealed a trend that showed lobular breast carcinoma might have the strongest association with Epstein-Barr virus infection. This meta-analysis showed a significant increase in breast malignancy risk in patients testing positive for the Epstein-Barr virus (OR = 6.29, 95% CI = 2.13–18.59). This result suggests that an Epstein-Barr virus infection is statistically associated with increased breast carcinoma risk.

## Introduction

Viruses are involved in the development of various cancers [1]. In 1995, the Epstein-Barr virus (EBV), an ubiquitous herpes virus, was found in 21% of 91 breast cancers [2]. Since then, a large number of studies have detected EBV infection in patients with breast carcinoma. A series of studies that adopted non-breast-cancer control groups have also been performed [2]–[11], and several mechanisms and hypotheses about the association between EBV infection and breast carcinoma have been developed [1], [12]–[17]. Some researchers believed that EBV infection may play a role in the early stages of breast carcinogenesis and elevate breast cancer risk [16]. Moreover, EBV infection might be a latent factor in the development of certain types of breast carcinoma [17]. However, statistical data from studies have varied widely. This inconsistency could be largely attributable to several problems: technical challenges in detecting and localizing the EBV in tumor cells, study designs that involved a specific histological type of breast carcinoma, and the lack of an epidemiological perspective that could clarify the inconsistencies in EBV prevalence across studies [18].

This study collected published information on EBV prevalence in breast carcinoma and explored the potential associations between EBV infection and breast cancer risk.

## Methods

### Searching and Selection

Medline and PubMed were searched for citations published from 1990 to September 2010 using the MeSH terms “Epstein-Barr virus,” “human,” “breast carcinoma” or “breast cancer”. Additional relevant references cited in retrieved articles were reviewed. Data extracted from studies had to meet the following criteria: (I) Studies had to use PCR-based techniques including quantitative PCR (QPCR) for detecting the EBV DNA in breast tissues. Studies based on cell lines or cells were not included, and the samples in the PCR assays could not have been preselected by other technologies such as immunohistochemistry or in-situ hybridization. (II) Data could be only on sporadic breast carcinoma. Studies in special groups were not included. (III) In instances where the research data were published in more than one article, only the publication with the most explicit description was included. (Figure S1)

### Data extraction

Data were extracted from each included study. Data included the first author's name, the journal, the year of publication, and the country of origin. Data were additionally extracted regarding the sample types, the PCR primers, and the EBV prevalence as detected by each kind of primer in each type of breast carcinoma tissues (if shown) by disease status and matching criteria if controls were present. To avoid bias from methods used in individual studies, only data acquired using PCR were used in the analyses.

### Study characteristics and quantitative data synthesis

Our meta-analysis consisted of three parts. The first part was an epidemiological description of the EBV prevalence in breast carcinoma tissues and an exploration of possible parameters for EBV infection or defection. The second part was a description of detecting the different amplification fragments in the EBV genome (*Bam H1W*, *Bam H1C*, *EBER2*, *LMP1*, *LMP2*, *EBNA1*, *EBNA4*, *BXLF1*, *BZLF1*, *BALF5*, *LF3*, *Gp220*, *Raji* and *Pol*) in the PCR assays. The third part was a statistical pooling of the EBV infection and breast malignancy risk estimates. The prevalence rate and the OR were the two main parameters for these studies.

An unconditional logistic regression model was adopted to compare and adjust the EBV prevalence by the following parameters: region (Asia, North Africa, North America, South America and Europe), sample types (fresh/frozen tissue or paraffin-embedded tissue), publication calendar period (1993–1999, 2000–2004 and 2005–2008), and the histological types. The effect of applying different PCR primers for detecting the EBV DNA in tissue samples was also evaluated. A McNemar test and Kappa test were adopted to estimate the diversity and consistency of the outcomes among the paired data extracted from available studies.

Fixed-effect and random-effect models were adopted to pool the patient and control data based on Mantel-Haenszel [19] and DerSimonian and Laird methods [20] according to the heterogeneity test from the studies. When heterogeneity is significant, a random-effects model is preferred. The heterogeneity test was performed using the *χ^2^*-based *I^2^* test and was considered significant when *p*<0.05. Begg's test and Harbord's weighted linear regression test [21] were used to evaluate the publication bias. Analyses were performed using STATA statistical software, Version 11.0.

## Results

### Epidemiological prevalence of the EBV in breast cancer

This analysis included 24 publications [2]–[4], [6]–[11], [22]–[35]. One of these publications [10] was divided into six parts to correspond to the six different countries or regions from where the cases were taken (information shown in Table S1). In the first analysis, 1535 cases from sixteen countries and regions were present (Table 1), with most cases taken from Europe (55.70%) and the Americas (20.33%). The prevalence of the EBV ranged from 0% to 53.58% in the selected individual studies, but the overall prevalence of the EBV in patients diagnosed with breast carcinoma was 29.32% (95% CI = 27.05–31.66%). The EBV prevalence was the lowest in the North American subjects (18.27%, 95% CI = 14.14–23.01%) and the highest in the Asian subjects (35.25%, 95% CI = 26.82–44.41%). Compared to the European subjects (the largest sample size), the North American subjects (USA) demonstrated a significantly lower prevalence of the EBV in breast cancer cases (OR = 0.48, 95% CI = 0.35–0.66 and OR = 0.62, 95% CI = 0.44–0.87 after adjustment). There were no significant differences in the EBV prevalence in breast cancer by the year of publication, although studies reported in the period 1993–1999 presented the highest prevalence (33.33%, 95% CI = 27.70–39.34). The prevalence of the EBV DNA was significantly lower (OR = 0.46, 95% CI = 0.35–0.61) when the samples were extracted from paraffin-embedded tissues (19.51%, 95% CI = 16.21–23.15%) compared with when they were extracted from fresh or frozen tissues (34.46%, 95% CI = 31.52–37.49%). Because there was no significant heterogeneity by calendar year or between the European and North African (P = 0.6900) populations, these factors were excluded and the EBV prevalence was recalculated to show that the EBV prevalence was lowest in the paraffin-embedded tissues from the North American subjects (10.71%, 95% CI = 6.47–16.40%, data not shown).

**Table 1:** EBV prevalence in all types of breast carcinoma cases.

Category	Subcategory	No. of studies	No. of cases	%	Prevalence (%)(95% CI)	Adjusted OR^c^
**Overall**	Total^a^	29	1535	100	29.32 (27.05–31.66)	-
**Region**	Europe	14	855	55.70	31.70 (28.59–34.93)	Ref
	North America (USA)	7	312	20.33	18.27 (14.14–23.01)	0.62 (0.44–0.87)
	South America	2	108	7.03	33.33 (24.55–43.05)	1.56 (0.98–2.47)
	Asia^b^	3	122	7.95	35.25 (26.82–44.41)	1.47 (0.91–2.40)
	North Africa	3	138	8.99	31.16 (23.55–39.59)	1.09 (0.73–1.62)
**Publication calendar period**	1993–1999	6	267	17.39	33.33 (27.70–39.34)	Ref
	2000–2004	14	850	55.38	28.35 (25.34–31.51)	0.79 (0.58–1.08)
	2005–2008	9	418	27.23	28.71 (24.42–33.31)	0.83 (0.56–1.23)
**DNA specimen**	Fresh/frozen tissue	15	1007	65.60	34.46 (31.52–37.49)	Ref
	Paraffin-embedded tissue	14	528	34.40	19.51 (16.21–23.15)	0.46 (0.35–0.61)

*95% CI:* confidence interval, *OR:* odds ratio.

a : One study [10] was divided into six parts because samples from six different countries were collected and tested.

b : Turkey was grouped in Asia because of its racial traits.

c : Adjusted by region, publication calendar period and DNA specimen.

**Figure 1:**
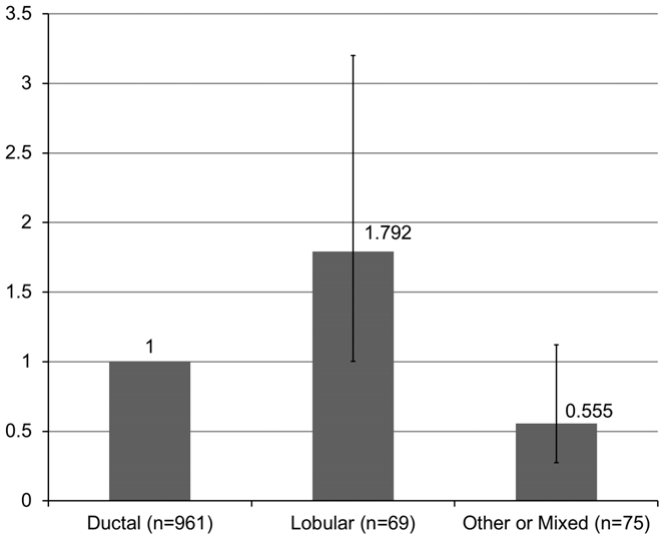
EBV DNA prevalence in specific types of breast carcinoma. Sixteen studies were selected and analyzed for the prevalence of EBV infection [2]–[4], [6], [7], [9]–[11], [22], [25], [26], [29], [30], [33]–[35]. The EBV prevalence rate by type of breast carcinoma is as follows: ductal (28.60%), lobular (34.78%) and other or mixed types (17.33%). Lobular carcinoma showed a significantly higher EBV prevalence than ductal carcinoma (adjusted OR = 1.792, 95% CI = 1.003–3.200, OR was adjusted by region, publication calendar period and specimen type), indicating that EBV infection probably increases breast carcinoma risk, especially for some specific types of carcinoma such as lobular carcinoma, according to available publications.

Sixteen studies were selected and analyzed for the prevalence of EBV infection. These studies offered clear information about the EBV prevalence in patients diagnosed with different histological types of breast carcinoma [2]–[4], [6], [7], [9]–[11], [22], [25], [26], [29], [30], [33]–[35]. Patients diagnosed with lobular breast carcinoma showed a higher prevalence of the EBV (Figure 1) than patients diagnosed with ductal carcinoma (adjusted OR = 1.792, 95% CI = 1.003–3.200).

### Different amplification fragments of EBV and breast cancer

Twenty one publications [2]–[10], [23]–[29], [31]–[35] that specified the individual DNA primers used for EBV detection were selected. One study [11] was excluded from this analysis due to the lack of information about respective prevalence when detecting different regions or genes in the EBV genome. One publication [10] was divided into six studies as described previously. The different primers used in the PCR assay to detect EBV DNA were summarized and analyzed. In total, 14 different genome fragments were amplified by respective primers (Table 2). The *Bam H1W* region (12 studies, 504 cases) was the most frequently used genome fragment for detecting EBV DNA in these studies and was used as the reference for normalizing and adjusting the incidence determined by the other fragments. The *Bam H1C* region (7 studies, 598 cases) was the next most frequently used fragment. *BXLF1* and *LF3* showed the highest prevalence rate of EBV in breast cancer, although this prevalence was based on a single study of only 15 cases. After adjustment, *BXLF1* demonstrated a significantly higher detection. *BZLF1*, *Raji* and *BALF5* showed a 0% prevalence and were included in only one study of approximately 50 cases. *EBER2* and *LMP1* were included in 3 studies with over 100 cases and showed significantly higher and lower detections of EBV, respectively. *Bam H1C* demonstrated a higher but significantly insignificant prevalence rate than *Bam H1W* (data shown in Table 2).

**Table 2:** Different primers used for detecting EBV prevalence in PCR analyses.

Amplification fragments	No. of studies^a^	No. of cases	Prevalence	Prevalence (%)(95% CI)	Adjusted OR^b^
*Bam H1W*	12	504	132	26.19 (22.40–30.26)	Ref
*Bam H1C* a	7	598	179	29.93 (26.29–33.78)	1.27 (0.83–1.93)
*EBER2*	3	141	50	35.46 (27.59–43.95)	1.74 (1.06–2.86)
*LMP1*	3	127	7	5.51 (2.24–11.03)	0.27 (0.12–0.59)
*EBNA1*	2	90	2	2.22 (0.27–7.71)	0.29 (0.05–1.64)
*BXLF1*	1	95	44	46.32 (36.02–56.85)	3.27 (1.79–5.98)
*Pol*	1	92	19	20.65 (12.92–30.36)	1.06 (0.55–2.05)
*Gp220*	1	57	13	22.81 (12.74–35.84)	0.15 (0.01–1.76)
*LMP2*	1	55	1	1.82 (0.05–9.72)	0.24 (0.03–2.18)
*BZLF1*	1	55	0	0 (0–6.49)c	0 (P = 0.0416)^d^
*EBNA4*	1	48	5	10.42 (3.47–22.66)	0.14 (0.04–0.47)
*Raji*	1	45	0	0 (0–7.87)^c^	0 (P = 0.0648)^d^
*BALF5*	1	45	0	0 (0–7.87)^c^	0 (P = 0.0648)^d^
*LF3*	1	15	6	40.00 (16.34–67.71)	2.53 (0.82–7.80)

*95% CI:* confidence interval, *OR:* odds ratio.

a : One study [10] was divided into six parts because samples from six different countries were tested. Three studies [11], [22], [30] were excluded because one [11] could not offer the EBV DNA prevalence detected by each primer, respectively, and the others [22], [30] only included 1 case.

b : adjusted by normalizing the constituent ratio of region and DNA specimen of the *Bam H1W* group.

c : one-sided, 97.5% confidence interval.

d : exact confidence levels not possible with zero count cells.

To further investigate the diversity and consistency among the prevalence outcomes for detecting different EBV genome fragments, six publications [2], [5], [8], [25], [29], [31] with paired data were selected for analysis (Table 3). *Bam H1W* is repeated 7 to 12 times in the EBV genome, making it a good target for detection of EBV DNA, even in a sample with a low viral copy number [5], [36], and our study supported this finding. *Bam H1C*, *EBER2* and *EBNA1* showed insignificant heterogeneity in EBV prevalence with *Bam H1W* and had a relatively higher breast cancer prevalence rate than *Bam H1W*. *LMP1* and *EBNA4* showed insignificant heterogeneity in detecting EBV prevalence and had significantly lower detection ability than *Bam H1W*. *BZLF1* demonstrated an insignificantly lower EBV detection ability while *LMP1* and *LMP2* showed significantly lower EBV detection abilities compared with *Bam H1W*. All of these results were consistent with our last analysis (Table 2), except the results from the targets with small sample sizes. A Kappa test showed that *Bam H1C* and *EBER2* displayed high and very high consistency with *Bam H1W* in the EBV detection. *LMP1* and *LMP2* demonstrated weak consistency, while *BZLF1* demonstrated no consistency with *Bam H1W* in the EBV detection. Meanwhile, *EBNA4* and *LMP1* demonstrated moderate consistency with each other (data shown in Table 3).

**Table 3:** Paired data of different detection fragments of EBV DNA.

Paired data of amplification fragments	No. of cases	Prevalence rate	Exact McNemar *P*	Agreement	Kappa	*Z* value	*P(>Z)*
*Bam H1W - Bam H1C*	89	21.35%–19.10%	0.6875	93.26%	0.7812	7.48	0.0000
*Bam H1W - EBER2*	108	33.33%–33.33%	1.0000	100%	1.0000	10.39	0.0000
*Bam H1W - LMP1*	79	11.39%–2.53%	0.0156	91.14%	0.3361	3.99	0.0000
*Bam H1W - EBNA1*	55	7.27%–3.64%	0.5000	98.18%	0.7909	6.00	0.0000
*Bam H1W - LMP2*	55	7.27%–1.82%	0.0156	94.55%	0.3820	3.60	0.0002
*Bam H1W - BZLF1*	55	7.27%–0%	0.1250	92.73%	0.0000	not available	not available
*LMP1 - EBNA4*	48	10.42%–10.42%	1.0000	91.67%	0.5535	3.83	0.0001

### Statistical pooling of EBV infection and breast cancer

**Figure 2:**
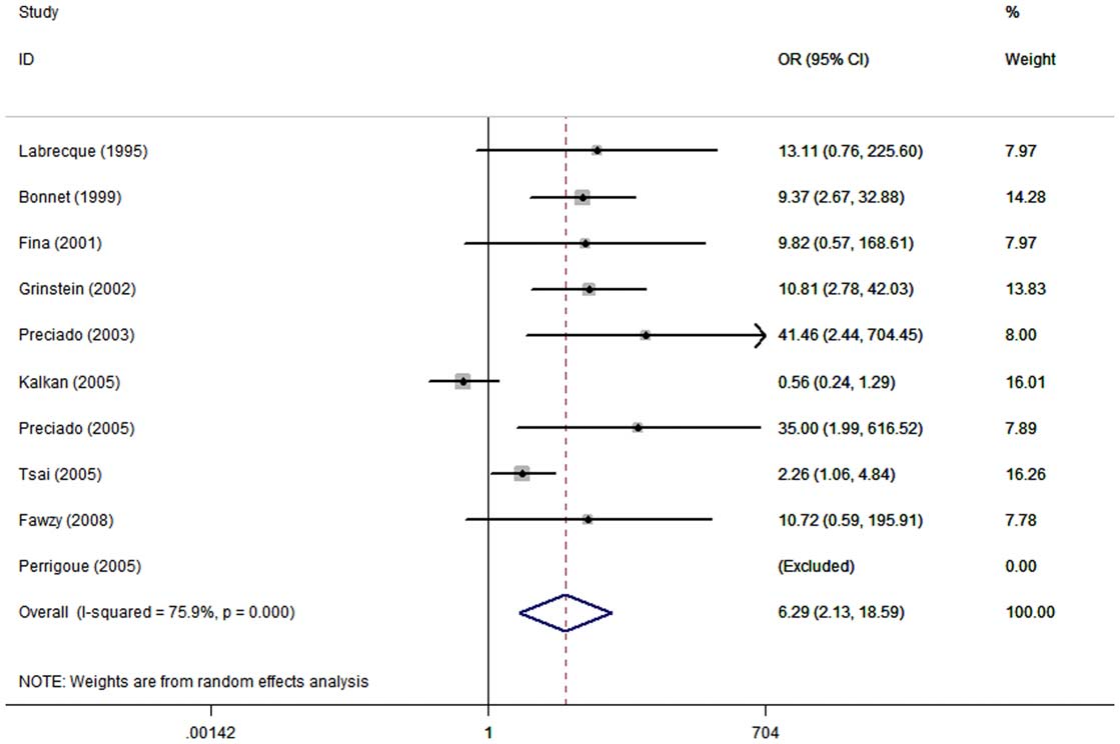
Association between EBV infection and breast carcinoma risk (ORs). Ten studies that adopted non-breast-cancer control groups [2]–[11] were selected for the analysis of EBV infection and human breast carcinoma risk. One [7] was excluded automatically after data pooling for the absence of EBV DNA in both patient and control groups. Random-effects model was chosen to estimate the ORs after pooling due to the high heterogeneity among these studies (*I^2^* = 75.9%, *P* = 0.000). A significant, 6.29-fold (95% CI = 2.13–18.59) increased breast carcinoma risk in patients with an EBV infection was shown.

**Figure 3:**
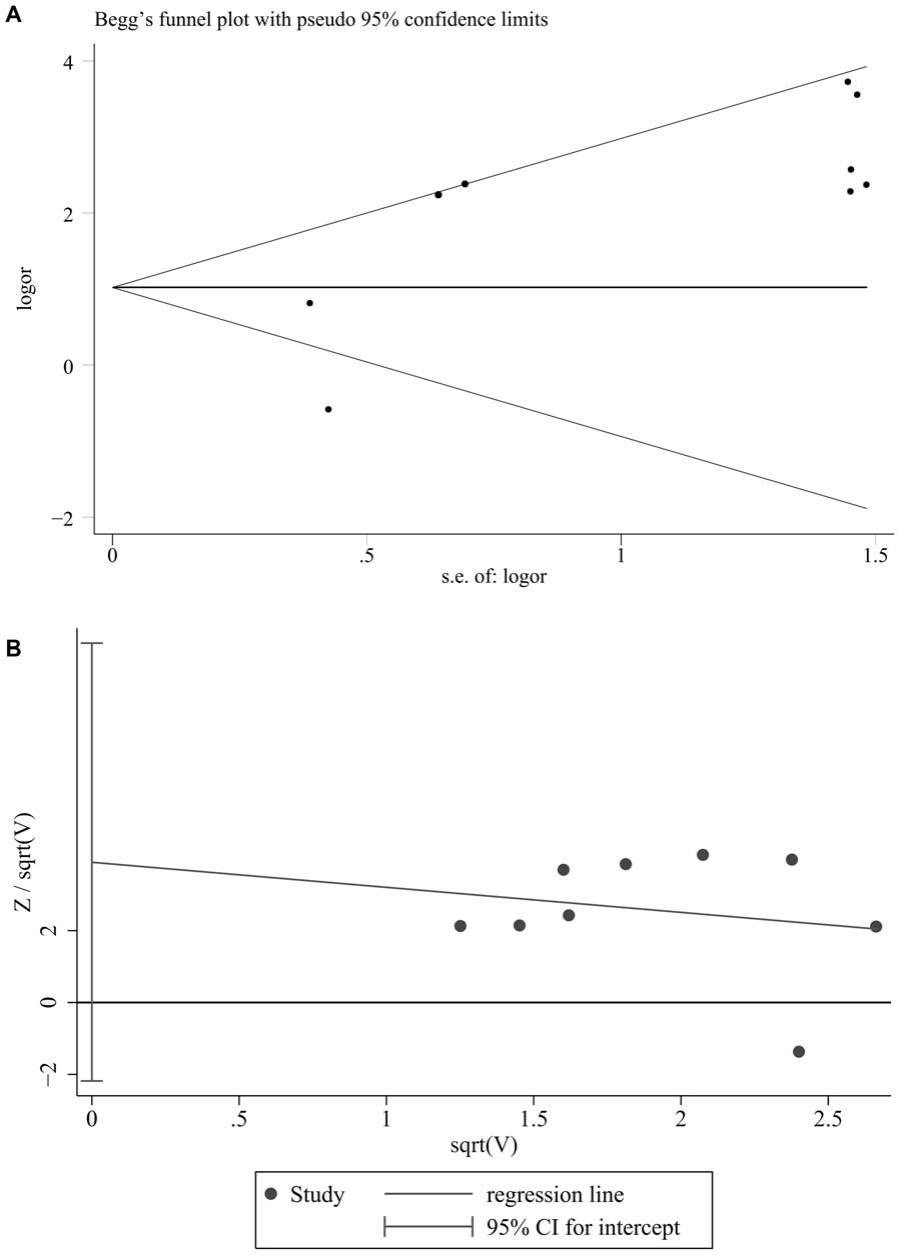
Estimating publication bias by *Begg's* test (A) and *Harbord's weighted linear regression* test (B). *Begg's* test (A) was adopted for measuring publication bias and showed a non-significant publication bias (*P* = 0.917). *Harbord's weighted linear regression* (B) also indicated a non-significant publication bias (*P* = 0.173).

Eleven studies included non-breast cancer control groups, one of which [27] was excluded because some of the control data were extracted from breast cancer cell lines. Therefore, 10 studies [2]–[11] were selected for this meta-analysis (detailed information is shown in Table S2), one of which [7] was excluded by the software after data pooling due to the absence of the EBV in both patient and control groups. The data analysis revealed high heterogeneity among these studies (*I^2^* = 75.9%, *P* = 0.000), so a random-effects model was chosen to estimate the OR after pooling (Figure 2). A 6.29-fold (95% CI = 2.13–18.59) increased breast carcinoma risk was associated with EBV infection (the OR values with a 95% CI from individual studies are shown in Table S3). For the sensitivity analysis, each study was excluded by turns. After that exclusion, depending on the heterogeneity among studies, fixed-effects or random-effects models were adopted for analyses. All the outcomes showed a significant association between EBV infection and increased breast carcinoma risk, as shown in Table S4, revealing a relatively low sensitivity. This low sensitivity suggests that our meta-analysis results are credible from a statistical perspective. To measure publication bias, a Begg's test and a Harbord's weighted linear regression test (Figure 3) were used, both of which were insignificant (*P* = 0.917 and *P* = 0.173, respectively).

## Discussion

Over the past few decades, the association between EBV infection and breast carcinoma has been debated. This debate is due, in part, to the epidemiological variation in EBV infections [16]. The more popular viewpoint is that this controversy might be due to differences in the methodologies or techniques used to detect the EBV in breast samples because different assays have different sensitivities and different standards for an “EBV positive” diagnosis. So far, polymerase chain reaction (PCR), immunohistochemistry (IHC) and *in situ* hybridization (ISH) are the most frequently used techniques to detect the EBV in breast tumors. Correspondingly, two general targets were used in detecting EBV in breast samples: a viral product, which includes the encoded RNAs and proteins, and the viral genome DNA itself.

EBV-encoded small RNAs (EBERs) *in situ* hybridization (EBER-ISH) is a highly sensitive method for detecting the EBV in breast tumor tissues. However, reports have indicated that EBV gene products, including many EBERs, may not be expressed detectably in EBV-positive tumor cells [9], [29], [37] because the targeted products are not expressed consistently in latent EBV infection or in certain stages of infection. Additionally, the possibility of an EBER-negative form of EBV infection has been presented [38], which could cause false-negative results and affect the accuracy of our analyses. Other reports showed that the EBER-1 transcripts only exist in a proportion of tumor cells, indicating the high expression of EBERs in infected cells might not be universal [2], [10], [29]. This result might additionally result in false-negative outcomes when the infected tissue blocks did not contain many EBER high-expression cells. In addition, false outcomes using *in situ* hybridizations (ISH) to detect EBERs may arise because low levels of expression of EBERs are nearly indistinguishable from background staining. The same weakness occurred when detecting the EBV by Immunohistochemical methods.

Immunohistochemistry (IHC) is also available for the detection of viral proteins, but some viral proteins are not consistently expressed in latent EBV infection. Though the EBV nuclear antigen-1 (EBNA1) is expressed in all forms of viral latency, as it is required for viral DNA replication and the maintenance of the EBV episome in infected cells [39], some antibodies used to detect EBNA1 produced only weak staining and could cross-react with other proteins, resulting in false outcomes from the IHC assays [27]. Thus immunohistochemical detection for EBNA1 is not reliable and these false outcomes could cause bias and affect the accuracy of our analyses.

The PCR method is a potentially important technique for detecting EBV DNA. A few studies that combined PCR with other detection methods such as IHC or EBER-ISH showed contradictory results [6], [28], [31]. The controversy may be a consequence of the different detection techniques. Nevertheless, many others studies showed significantly correlated results [3], [5], [8]–[11], [30] and recommended the simple PCR method as the first priority for detecting the EBV in breast tissues.

In light of the merits and drawbacks of each method, we chose statistical data from studies which detected EBV DNA with PCR techniques to identify the correlation between EBV infection and breast cancer risk.

Our study showed a significant heterogeneity of EBV prevalence (OR = 0.62, 95% CI = 0.44–0.87) between Europe and the USA in breast carcinoma tissues, which indicated that there were fewer associations between EBV infection and breast carcinoma cases in the USA than in Europe. Reasons for this difference are unclear but may be related to the prevalence of the EBV and differences in testing methods and diagnostic criteria for EBV infection in the USA. In addition, the potential influence of a multi-cultural society and large number of migrants may play a role in explaining this difference. The EBV prevalence in breast carcinoma cases was slightly higher in the 1993–1999 publication period (33.33%, 95% CI = 27.70–39.34%) and then dropped to 28.35% in 2000–2004 and rose slightly to 28.71% in the last period. These variations were insignificant. The decrease from 2000–2004 may indicate more stable data because of larger sample sizes (850 cases versus 267 cases in 1993–1999). The increase after 2004 was probably related to the technical improvements in EBV DNA detection. EBV detection in fresh/frozen tissue showed many more positive results (34.46%) than in paraffin-embedded tissue, and the heterogeneity was statistically significant (OR = 0.46, 95% CI = 0.35–0.61), indicating that a biopsy or different slide preparation of tested specimens may yield different results. and break DNA strands. Moreover, it is much simpler paraffin-embedded for DNA detection. However, we did not find paraffin-embedded tissue and fresh/frozen tissue for the same patients, so it is difficult to conclude Normally frozen tissues samples show higher “fidelity” than paraffin-embedded tissue because some necessary processes and chemicals used to prepare paraffin-embedded samples, such as xylene, could damage whether fresh/frozen samples are more suitable for EBV detection than paraffin-embedded tissue.

We collected information about the EBV prevalence in different types of breast carcinomas with a prevalence rate of 28.60 in ductal, 34.78% in lobular, and 17.33% in other or mixed type breast carcinomas. The statistical analysis revealed that lobular breast carcinoma had a higher correlation with the EBV than did ductal carcinoma (adjusted OR = 1.792, 95% CI = 1.003–3.200), though it is unclear whether this significant difference results from biological patterns. Both lobular and ductal breast carcinoma show a relatively higher EBV prevalence than mixed carcinomas or other types of breast tumors. These differences in prevalence may be because EBV infection elevates breast cancer risk in some specific types of breast carcinoma [17]. Invasive lobular and ductal breast cancers may have a higher association with the EBV [2].

Fourteen different EBV DNA regions or genes were detected in breast carcinoma tissues (Table 2) and demonstrated various detection abilities for the EBV. *Bam H1W* was adopted in 12 studies as a reference. *Bam H1C* showed similar detection rate to *Bam H1W* (OR = 1.27, 95% CI = 0.83–1.93), both revealed a relatively high EBV prevalence (26.19% and 29.93%, respectively) and both of them included over 500 cases, providing relatively stable results. In this analysis, *BZLF1*, *Raji* and *BALF5* showed a 0 detection rate, but the heterogeneity is not significant compared with *Bam H1W*. These results support *Bam H1W* as a good biomarker for EBV due to the significance of these genes, such as *BZLF1*, which is a lytic gene rather than a latent gene. For further analysis, paired data were extracted from six publications and analyzed by a McNemar test and a Kappa test (Table 3). The outcomes were consistent with the former result, indicating that *Bam H1W*, *Bam H1C* and *EBER2* were preferred markers for EBV detection in breast carcinoma tissues used in the PCR assays. This analysis indicates the importance of the PCR target on the measured extent of the association of the EBV with breast carcinoma [40].

The potential relationship between breast cancer and EBV infection is important because this relationship can not only broaden our understanding of breast cancer etiology, but also be applied to early detection of breast cancer [15], as well as breast cancer prevention and treatment [41]. By pooling the data published from 1990 to 2010, our study reported the EBV prevalence rate in breast carcinoma tissues and analyzed certain parameters related to detecting EBV prevalence in breast cancer tissues.

To the best of our knowledge, this is the first meta-analysis to test the association between EBV infection and breast carcinoma. Moreover, potentially important parameters were evaluated in this study, including the geographical region, the publication calendar period, the DNA specimens, the histological types of breast carcinoma and the detection regions or genes in EBV genome, which could affect EBV detection in breast carcinoma tissues [6], [10], [23]. Our study focuses only on research that tested the presence of viral particles and does not include immunological evidence of past or present infection because those studies are too few to perform an analysis and, in some, the methodology did not meet our matching criteria [42]. In the future, more attention should be paid to the immunological evidence of past or present EBV infection because it may contribute to our understanding of the relationship between EBV infection and breast cancer risk.

Moreover, the cellular location of the EBV in breast cancer has become important because EBV-positive infiltrating lymphocytes might partly explain the presence of the EBV in breast tumors that show a positive result by PCR but a negative result by ISH or IHC. Efforts have been made to identify the cellular location of EBV genetic material in breast cancers. Researchers adopted microdissection to isolate cancer cells from breast stroma cells and lymphocytes, and applied ISH and IHC combined with PCR to resolve this problem. Reports have shown that EBV DNA has a higher prevalence in cancer cells than in infiltrating lymphocytes, and only a proportion of breast cancer tissues test positive for the EBV [2], [10], [11], [24], [26]. Preciado et al. also showed that their EBV positive result assumed that the cellular source of the PCR EBV signal came from the epithelial tumor cells because the bystander lymphocytes of stroma were negative [8]. Alternatively, studies using laser capture microdissection combined with RT-QPCR revealed that EBV genomes were heterogeneously distributed in morphologically identical tumor cells, with some isolated tumor cells testing negative for EBV-DNA while other clusters of isolated tumor cells from the same specimen contained relatively high genome numbers [24].

### Limitations

Though our study found a positive correlation between the EBV and breast cancer, the presence and implication of EBV infection in the initiation and progression of breast cancer remains controversial. The conflicting results may be explained by the use of different technical approaches for detecting the EBV. Certain technique groups failed to detect the EBV [28], [33], [35], [43]–[46], whereas others showed positive results depending on the methodology applied. For instance, 2 PCR-EBV-negative samples were stained by EBNA1 MAb in Fawzy's research [3]. Murray et al. failed to detect the EBV genome by QPCR though these samples were EBV positive based on their immunochemistry [27]. The dissimilarities in the sample types (fresh/frozen tissue or paraffin-embedded tissue), study population (Asian, European, American, etc.) and heterogeneity among cluster cells could contribute to this discrepancy. The technical limitations of the assays were associated with the publication calendar period and may result in inconsistent outcomes. More studies in this area are needed to clarify the reasons behind these apparently conflicting outcomes.

We found only two studies that adopted control groups [6], [7] and matched our selection criteria, of which one [7] was excluded automatically by the software when we performed meta-analysis. Though the Begg's test and Harbord's weighted linear regression test proved the publication bias was non-significant, the only study [6] showing the opposite trend of the other studies might reflect the difficulties of publishing “negative” results.

Another problem is that though EBV detection in fresh/frozen tissue showed statistically higher prevalence ratio than in paraffin-embedded tissue (OR = 0.46, 95% CI = 0.35–0.61), we did not find paraffin-embedded tissue and fresh/frozen tissue matched for the same patients. Therefore, it is difficult to determine whether fresh or frozen tissue is more suitable for EBV detection. Future studies that adopt paraffin-embedded tissue and fresh/frozen tissue matched for the same patients, especially for samples have been stored for a long period of time, will help us to resolve this problem and help to understand optimal the conditions for detecting the EBV in breast cancer tissue.

Our study indicated that an EBV infection is statistically associated with an increased breast carcinoma risk, especially for some specific types of carcinoma, such as lobular carcinoma, according to available publications. EBV infection may play a role in breast cancer oncogenesis and may result in a more aggressive phenotype, which is supported by the relative aggressiveness of EBV-associated breast carcinomas when compared with non-EBV-associated breast carcinomas [11], [27]. A recent study [47] that use RT-QPCR as a detection technique concurred that EBV-positive breast cancers presented a more aggressive phenotype. In this study, there was a difference between EBV-positive and EBV-negative breast cancers in clinical and biological profiles, and EBV-positive samples represented a higher proportion among the high-grade and ER-negative breast cancers. Whether EBV is a primary etiological agent is unknown. Additional studies, especially those that include paired non-breast-cancer control groups and contain larger scale random samples, are needed to achieve more consistent conclusions regarding the association between EBV infection and breast carcinoma risk.

## Supporting Information

Figure S1 **Flow of involved studies.**
(TIF)

Table S1 **Detail information and list of extracted studies for general analyses.**
(XLS)

Table S2 **Detail information and list of extracted studies for meta-analysis.**
(DOC)

Table S3 **Primary results of individual studies adopted in the meta-analysis.**
(DOC)

Table S4 **Sensitivity analysis after each study was excluded by turns.**
(DOC)
