# Supplementary figures and images for: Epstein-Barr Virus Infection and Sporadic Breast Cancer Risk: A Meta-Analysis
Source: PLoS One. 2012 Feb 21;7(2):e31656. doi: 10.1371/journal.pone.0031656 (PMC3283657; doi:10.1371/journal.pone.0031656)

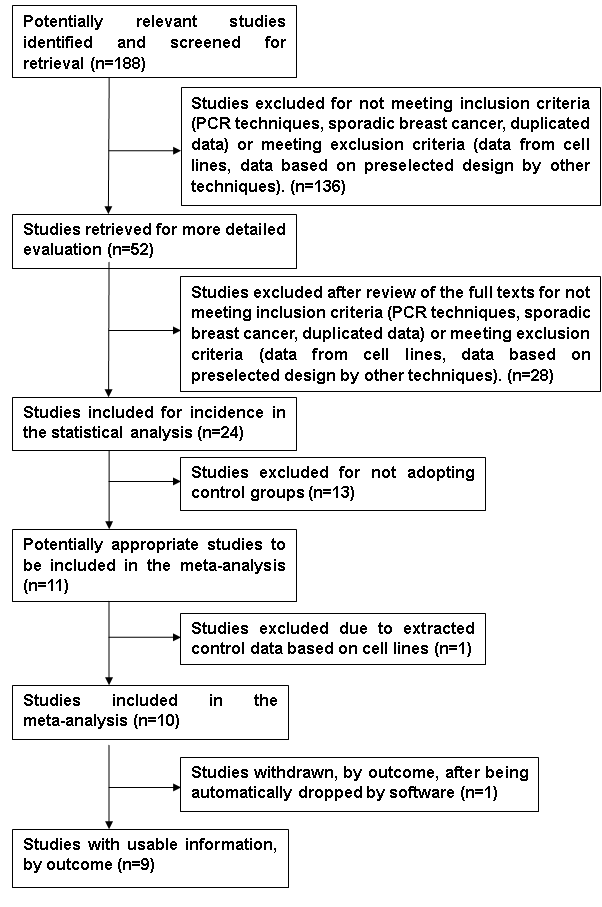

Supplement: Figure S1 — Flow of involved studies. (TIF) [file pone.0031656.s001.tif]
