# Supplementary material for: Epstein-Barr Virus Infection and Sporadic Breast Cancer Risk: A Meta-Analysis
Source: PLoS One. 2012 Feb 21;7(2):e31656. doi: 10.1371/journal.pone.0031656 (PMC3283657; doi:10.1371/journal.pone.0031656)
Supplement: Table S2 — Detail information and list of extracted studies for meta-analysis. (DOC) [file pone.0031656.s003.doc]

**Table S2.** Detail information and List of extracted studies for Meta-analysis.

| **Study** | **Patient Groups** | **Control Groups** |
| --- | --- | --- |
| [Labrecque *et al*, 1995](#_ENREF_6) | 19/91 Breast carcinoma samples | 0/20 Benign breast tumor; 0/1 normal breast tissues |
| [Bonnet *et al*, 1999](#_ENREF_1) | 51/100 consecutive primary invasive breast carcinoma tissues | 3/30 healthy tissues adjacent to a subset of the tumors |
| [Fina *et al*, 2001](#_ENREF_3) | 162/509 primary invasive ductal breast cancer tissues | 0/7 benign breast tumor tissues; 0/3 normal tissues adjacent to breast cancers |
| [Grinstein *et al*, 2002](#_ENREF_4) | 8/20 infiltrating ductal breast carcinoma; 6/13 infiltrating lobular carcinoma | 0/21 normal breast tissues; 2/16 non-proliferative variants tissues of fibrocystic changes; 1/10 benign fibroadenoma tissues |
| [Preciado, 2003](#_ENREF_8) | 24/69 breast carcinoma epithelial tissues | 0/30 benign breast tumor biopsies with no atypia; 0/8 normal breast tissues |
| [Kalkan *et al*, 2005](#_ENREF_5) | 7/28 Invasive ductal BC; 4/20 invasive lobular BC; 2/9 carious malignant lesion samples | 19/55 normal breast tissues or benign breast diseases samples (including 34 fibrocystic disease samples, 16 fibroadenomatous tissues, 5 various benign lesion/normal breast samples) |
| [Perrigoue *et al*, 2005](#_ENREF_7)  (Automatically excluded) | 0/37 infiltrating ductal BC; 0/4 infiltrating lobular BC; 0/3 tubular carcinomas; 0/1 mucinous carcinoma | 0/45 matched normal samples |
| [Preciado *et al*, 2005](#_ENREF_9) | 12/39 fresh breast carcinoma tissues | 0/30 biopsy specimens of benign breast tumors with no atypia; 0/8 matched normal breast tissues |
| [Tsai *et al*, 2005](#_ENREF_10) | 28/62 invasive ductal breast cancer specimens | 0/12 non-cancerous group (including normal, fibrosis, or breast fibrocystic changes); 16/32 benign mammary fibroadenoma; 0/16 benign thyroid tumor specimens |
| [Fawzy *et al*, 2008](#_ENREF_2) | 4/32 invasive ductal BC; 4/8 invasive lobular BC | 0/20 matched fibrocystic disease tissues |
