# Supplementary material for: Epstein-Barr Virus Infection and Sporadic Breast Cancer Risk: A Meta-Analysis
Source: PLoS One. 2012 Feb 21;7(2):e31656. doi: 10.1371/journal.pone.0031656 (PMC3283657; doi:10.1371/journal.pone.0031656)
Supplement: Table S3 — Primary results of individual studies adopted in the meta-analysis. (DOC) [file pone.0031656.s004.doc]

**Table S3.** Primary outcomes of each study which was adopted in Meta-analysis.

| **Study** | **OR (95% Conf. Interval)** | **% Weight** |
| --- | --- | --- |
| [Labrecque *et al*, 1995](#_ENREF_6) | 13.113 (0.762-225.601) | 7.97 |
| [Bonnet *et al*, 1999](#_ENREF_1) | 9.367 (2.669-32.878) | 14.28 |
| [Fina *et al*, 2001](#_ENREF_3) | 9.820 (0.572-168.608) | 7.97 |
| [Grinstein *et al*, 2002](#_ENREF_4) | 10.807 (2.779-42.025) | 13.83 |
| [Preciado, 2003](#_ENREF_8) | 41.462 (2.440-704.451) | 8.00 |
| [Kalkan *et al*, 2005](#_ENREF_5) | 0.560 (0.244-1.286) | 16.01 |
| [Preciado *et al*, 2005](#_ENREF_9) | 35.000 (1.987-616.522) | 7.89 |
| [Tsai *et al*, 2005](#_ENREF_10) | 2.265 (1.059-4.842) | 16.26 |
| [Fawzy *et al*, 2008](#_ENREF_2) | 10.723 (0.587-195.907) | 7.78 |
