# Supplementary material for: Epstein-Barr Virus Infection and Sporadic Breast Cancer Risk: A Meta-Analysis
Source: PLoS One. 2012 Feb 21;7(2):e31656. doi: 10.1371/journal.pone.0031656 (PMC3283657; doi:10.1371/journal.pone.0031656)
Supplement: Table S4 — Sensitivity analysis after each study was excluded by turns. (DOC) [file pone.0031656.s005.doc]

**Table S4.** Sensitivity analysis after each study was excluded by turns.

| **Excluded Study** | **Heterogeneity** | **Overall OR (95% CI)** |
| --- | --- | --- |
| [Labrecque *et al*, 1995](#_ENREF_6) | Significant (*I^2^* = 78.0%, *P* = 0.000) | 5.94 (1.90-18.56) |
| [Bonnet *et al*, 1999](#_ENREF_1) | Significant (*I^2^* = 76.2%, *P* = 0.000) | 6.02 (1.80-20.15) |
| [Fina *et al*, 2001](#_ENREF_3) | Significant (*I^2^* = 78.3%, *P* = 0.000) | 6.11 (1.94-19.27) |
| [Grinstein *et al*, 2002](#_ENREF_4) | Significant (*I^2^* = 76.2%, *P* = 0.000) | 5.87 (1.78-19.36) |
| [Preciado, 2003](#_ENREF_8) | Significant (*I^2^* = 75.6%, *P* = 0.000) | 5.23 (1.76-15.52) |
| [Kalkan *et al*, 2005](#_ENREF_5) | Non-significant (*I^2^* = 76.2%, *P* = 0.000) | 7.02 (4.24-11.63) |
| [Preciado *et al*, 2005](#_ENREF_9) | Significant (*I^2^* = 42.3%, *P* = 0.096) | 5.94 (1.90-18.56) |
| [Tsai *et al*, 2005](#_ENREF_10) | Significant (*I^2^* = 76.4%, *P* = 0.000) | 5.36 (1.78-16.15) |
| [Fawzy *et al*, 2008](#_ENREF_2) | Significant (*I^2^* = 78.4%, *P* = 0.000) | 8.36 (2.06-33.97) |
